# What makes the pregnant women revisit public hospitals for research? Participant engagement and retention trial in a public hospital (PERTH): an RCT protocol

**DOI:** 10.1186/s12884-018-2000-1

**Published:** 2018-09-12

**Authors:** Giridhara R Babu, Maithili Karthik, Deepa Ravi, Yamuna Ana, Prafulla Shriyan, Kiran Kumar Hasige, Keerti Deshpande, Lokesh Bangalore Siddlingaiah, Sanjay Kinra, Gudlavalleti Venkata Satyanarayana Murthy

**Affiliations:** 1 Public Health Foundation of India (PHFI), IIPH-H, Bengaluru campus, SIHFW premises, Beside leprosy hospital, 1st cross, Magadi road, Bengaluru, 560023 India; 2 Jayanagar General Hospital, Bengaluru, India; 3 London School of Hygiene & Tropical Medicine & University College London Hospital, London, UK; 4 Indian Institute of Public Health-Hyderabad, Plot # 1, A.N.V.Arcade, Amar Co-op Society, Kavuri Hills, Madhapur, Hyderabad, 500033 India; 5 London School of Hygiene & Tropical Medicine, ICEH, 3rd Floor, South Courtyard, Keppel Street, London, WC1E 7HT UK

**Keywords:** Birth cohort, Interactive voice response system, Follow up, Public hospital, Health communication, New technologies, Care management, Cost-effective

## Abstract

**Background:**

Cohort studies have public health importance as they effectively provide evidence on determinants of health from a life course perspective. Researchers often confront the poor follow-up rates as a major challenge in the successful conduct of cohort studies. We are currently recruiting in a birth cohort study, titled as “Maternal Antecedents of Adiposity and Studying the Transgenerational role of Hyperglycemia and Insulin” (MAASTHI) in a public hospital; with the aim of assessing maternal glycemic levels on the risk of adverse fetal outcomes. Nested within the ongoing cohort, the proposed trial aims to evaluate the effectiveness of two interventions in improving the follow-up in the cohort study in a public hospital.

**Methods:**

A randomized trial of 795 pregnant women, with 265 women each in three arms observed through pregnancy, until their baby is 14 weeks old. The comparator group receives a standard leaflet, with details on the importance of glucose testing and regular follow up in pregnancy. Intervention arm-1 will receive the standard leaflet plus individualized messages, through an Interactive Voice Response (IVR) system; a type of computer-linked telephone intervention system to remind the participants about the lab test and follow-up dates. Intervention arm- 2 will have the opportunity to attend Mother and Baby Affairs (MBA) workshops, which will provide information on Gestational Diabetes Mellitus (GDM) screening and management to pregnant women and personalized counselling services. The outcome of interest is the difference in the proportion of participants completing follow-up at different points in time, among three arms.

**Discussion:**

Between the two interventions (IVR and MBA), the study results would uncover the contextually specific, timely intervention, which can increase the proportion of pregnant women followed up in public hospitals. If effective, this study will provide information on an effective intervention, useful in ensuring the success of longitudinal follow-up in the public hospitals.

**Trial registration:**

NCT03088501, Date Registered: 16/03/2017.

## Background

Longitudinal cohort studies are essential in understanding the etiological mechanisms of underlying hypotheses. It is particularly challenging to develop and sustain birth cohorts, as the observation period spans several years making it vulnerable to lose tracking subjects for several reasons. These include a change in the location of residence, lack of interest in the study, death etc. [1, 2]. Few groups are at higher risk of selective attrition, particularly ethnic minorities, those with low family income or low education, and those living in urban areas [3].

The results from prospective cohort studies can be unbiased, only if they can maintain greater compliance of follow-up. The loss to follow-up can result in biased estimates [4], if the reason for the loss is related to the outcome of the investigation. The cohort studies done in India have variable rates of loss to follow-up ranging from 14 to 82% [1, 2, 5, 6]. Continuous follow-up and ensuring retention of participants is challenging due to many reasons. These include lack of trust in the research, concerns about research design, the consent process, discordance between lay beliefs, medical practice and cost of travel, etc. This challenge is intricate given that pregnant women accessing public health facilities do not turn up for regular antenatal health checkups [7]; which are influenced by limited decision making power regarding their health, poor access, transport for routine care, perceived the poor quality of health care facilities and limited staff/financial constraints [8]. According to the National Family Health Survey (NFHS) (2014–15), only 55.5% of women in urban Bengaluru had the required four antenatal care visits during pregnancy [9].

Some reviews have reported different strategies like using a mobile phone, Interactive voice response, antenatal sessions and community-centered approaches for improving study participation and retaining a greater proportion of participants [10–17]. For example, an automated voice call was used to improve adherence to iron supplementation during pregnancy [10]. However, little is known about the effectiveness of specific retention strategies deployed in retention of mothers and infants in a birth cohort.

MAASTHI is a prospective birth cohort with the aim of assessing the effects of glucose levels in pregnancy on the risk of adverse infant outcomes, especially in predicting the possible risk markers of later chronic diseases [18]. The recruitment of the pregnant women in MAASTHI began in April 2016. The current strategy deployed in MAASTHI cohort is to follow up pregnant women for Oral Glucose Tolerance Test (OGTT), at birth and in-person child assessments at 14 weeks, annually during the year 1, 2 and 3 of the child. Currently, we are recruiting around 80 pregnant women per month, with the completion of all their records, anthropometry measurements, and OGTT. Of the eligible pregnant women, only 77% completed OGTT despite offering the test free of cost and repeated reminders by the research team to get tested for gestational diabetes. Once the OGTT is done between 24 and 36 weeks of gestation, there is no further opportunity to meet them again until the delivery. The duration without contact increases the likelihood of attrition. In the intervening period of initial contact and follow-up’s, it is essential that the participants are continuously engaged by the research staff through innovative methods in order to attain better turn up rate for follow up. Despite stringent adherence of including only the residents of the source population, nearly 20% of the women did not attend the follow-up visits at birth. In order to prevent further loss to follow-ups in subsequent visits, we are interested in exploring whether interventions involving Interactive voice response system (IVRS) and conducting mother and baby workshops can improve follow-up rates.

## Methods

The primary objective of the RCT is to evaluate the effectiveness of two interventions; IVRS and mothers and baby affairs (MBA) workshops in improving the response rate of participants for OGTT. The secondary objective is to evaluate the effectiveness of interventions in improving the follow-up rates of participants in the cohort and to also identify process indicators which contribute to observed changes in follow-up compliance and to estimate the cost-benefit analysis of the interventions offered in the study.

### Study setting

The study is conducted in a secondary level public hospitals in urban Bengaluru.

### Study population

The study population comprises of pregnant mothers in the ongoing MAASTHI cohort. By recruiting the pregnant women attending Antenatal Checkup (ANC) in a public hospital, participants will be randomly assigned to the MBA or IVRS arm or continued in the control group.

#### Inclusion criteria

- Pregnant women aged 18–45 years
- Pregnant mothers of 14 to 36 weeks of gestational age
- Agree by providing informed consent to take part in the IVRS/MBA workshop intervention.
- She must be able to speak Kannada, Hindi or English.
- The participant must own or have sufficient access to a cell phone and should be able to operate a cell phone with a partner/ relative, who stay with her.

#### Exclusion criteria

- Pregnant mothers with a history of Diabetes or Hepatitis B infection;
- Human Immunodeficiency Virus (HIV) positivity;
- Pregnant women of other gestational ages

##### Study design

**Fig. 1:**
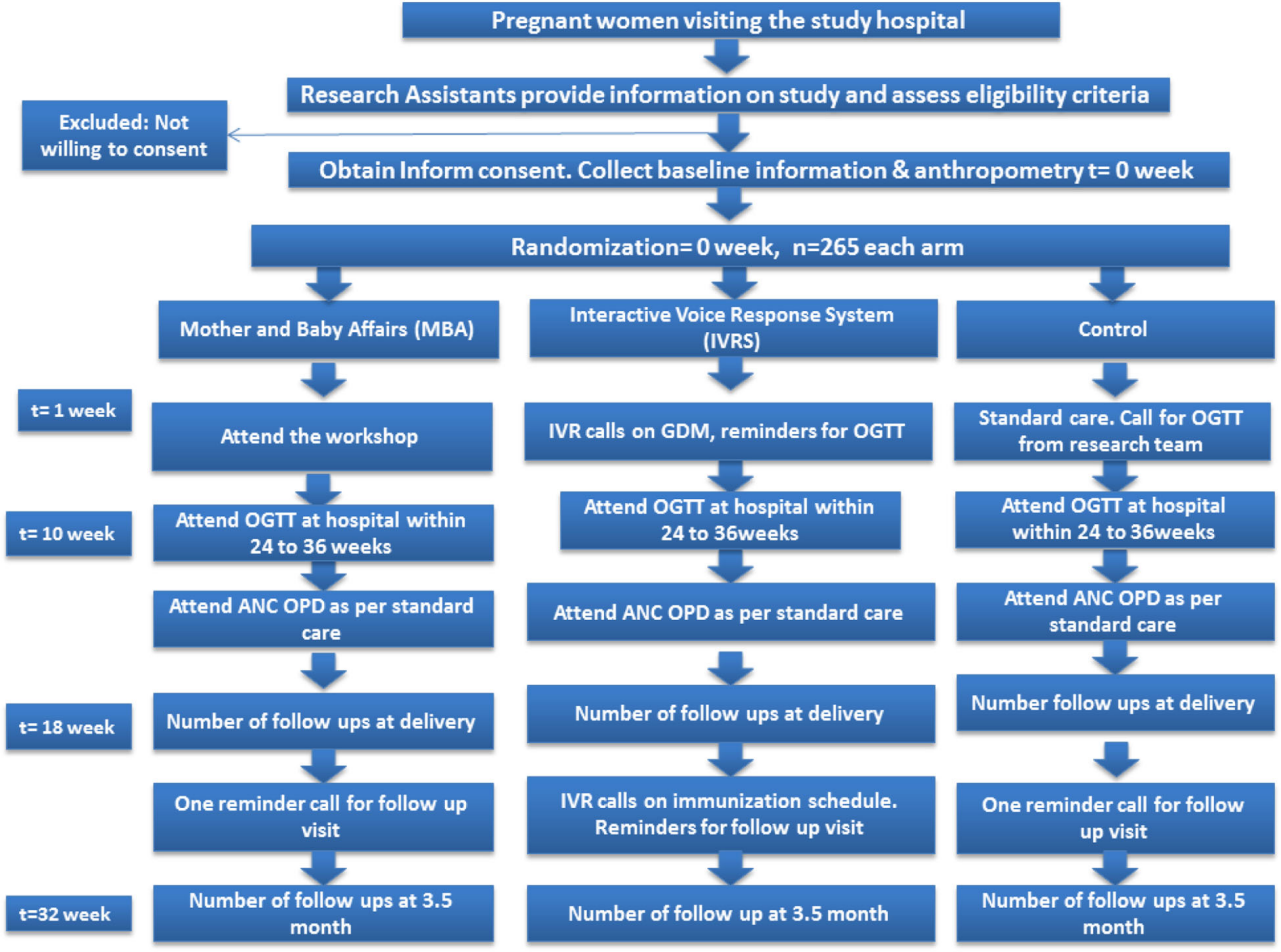
Flowchart depicting the study design and allocation of participants to different study arms

The prospective study subjects of MAASTHI cohort will undergo a Randomized Controlled Trial (RCT). With the aid of a random number generator, the participants will be randomized into each arm on a 1:1:1, using a computer-generated randomization list. A parallel group design superiority trial; the sequence generation and allocation concealment will not be involved in the implementation (Fig. 1).

### Blinding

After meeting the inclusion criteria, voluntarily providing informed consent, the research assistants will assign the participants to one of three study arms. Once the participant is assigned to the arm, the research staff will be recording her unique respondent identification number and the assigned arm. Research staff and pregnant women cannot be blinded because the intervention requires their participation; however, the data analyst would remain blind to the study arm assignment.

### The intervention

#### Theoretical background

The proposed RCT is evidence as well as theory based and follows a planned stepwise behavioural research approach to intervention development [19]. The self-regulation theory suggests that change in behaviour is a dynamic process, in which specific, achievable goals are set, and discrepancies between desired goals is fed back to an individual, and finally, (social) reinforcement as an essential element promotes behavioural sustainability and ongoing goal pursuit [20, 21]. The approach focuses on individual-level factors, increasing personal and normative feedback. Among others, it addresses these elements using increasing planning, performance feedback, and increasing knowledge. Self-regulation theory focuses primarily on a behavioural prediction; self-regulation models attempt to explicate the dynamic psychological mechanisms that lead to success and failure in behaving relative to some standard. Intervention package includes changes to the physical and social environment, performance feedback & goal setting, training and action planning.

The interventions are:

- IVRS intervention- interactive voice response.
- MBA intervention-Mother and baby affairs
- Control arm

The two proposed arms in the intervention, namely IVRS and Mother and baby affairs (MBA) workshops are designed to set goals with options for feedback to an individual; while the use of technology for (social) reinforcement is done in one arm, the other arm has peer pressure and discussion on shared goals. The interventions are expected to promote and sustain behavioural changes.

##### Interactive voice response (IVR):

IVRS are a type of computer-linked telephone intervention system that could be used to remind the participants about their medicine, treatment or follow up visit. IVR can provide individualized messages to participants and obtain feedback from participants’ responses through voice recognition or touch-tone keypad. IVR systems have shown potential for use as a tool in health care. For example, IVRS as a reminder system helped to increase preventive screening and vaccinations [22], as a means for screening high-risk pregnant women for depression [23]. IVR allows the pregnant women, their family members to interact with a computer system using a telephone. IVR systems are ubiquitous and are used for banking, flight schedules, and product ordering. Socioeconomically disadvantaged populations react favourably to and use IVR systems for extended durations [24]. Studies show that those with a lower income similar to that of our population are more likely to use IVR [25]. Through IVR, women receive a 2–3 min call that informs them about OGTT test dates, to collect their lab test reports, to know where they plan to deliver and reminds them of their follow-up visits. The IVRS will also provide health information on Gestational Diabetes Mellitus (GDM), breastfeeding and immunization. Research suggests that some socially disadvantaged populations are more likely than their advantaged counterparts to text daily [26]. Telephone technologies have an extensive reach, as mobile phone penetration is very high in the target population. In our study population, 58.6% of the women own cell phone, and everyone has at least one cell phone in their family. If the respondent does not wish to continue in the IVRS arm, they will have option to move out of this arm and continue in the control arm.

The control arm will not get the interventions but would receive a standard leaflet, with details on the importance of glucose testing and regular follow up in pregnancy; research staff would inform them the next visit date. They will be asked to meet the research team whenever they visit the hospital for their routine checkups. At each visit, the research staff will assess their general well-being and other health parameters. They will also provide standard information on health and the importance of continuing antenatal examination. If any of the interventions in the other two arms are proven successful, the control participants will receive those interventions for the subsequent follow-ups.

##### Mother and baby affairs (MBA) workshops:

This arm of intervention will involve antenatal workshop and counselling for parents. This will include organizing a lunch for the participant and her family (husband and kids if any). Generally, this is called as “Seemantha” in Kannada similar to “Godhbharai” in northern India and baby shower in other parts of the world. However, the intervention will involve particular components. Such as

- A brief talk by health professionals on importance of GDM screening and management and other antenatal and postnatal topics.
- A health-related quiz competition followed by gifts distribution to winners and
- Role-plays on personal hygiene, the importance of having nutritional food, delivering at the hospital, precautions at the time of delivery, caring for the baby, feeding of colostrum, homemade baby food, immunization, and family planning measures.

Conducted ahead of OGTT, this activity will be done every month in batches for around 25 participants. We will also ensure father’s participation in the program for increased engagement in managing the health of mother and child. Anganwadi Worker (AWW) and Accredited Social Health Activist (ASHA) workers will also be involved.

### Implementation

The research staff will inform the study participants of MAASTHI birth cohort regarding the interventional trial. Upon voluntary informed consent, the research staff will screen the participants for eligibility and will randomly allocate to one of the study arms. Necessary demographic details, socioeconomic status, diet, physical activity are recorded. Participants will be randomly allocated to the study arms-, IVRS arm, MBA arm or control arm. They are invited to undergo the OGTT between 24 to 36 weeks of gestation. A portion of the blood sample will be stored at − 80 degrees for future biochemical and genetic tests.

In addition to the standard care, the MBA arm will receive intervention in the form of awareness workshop from specialist doctors. There will be role plays and quizzes, participants who win will receive small gifts from the study team.

Along with standard care, the IVRS intervention involves calls to pregnant women at specified periodic time intervals providing them with antenatal care tips, and reminder calls for OGTT and follow-up visits to the hospital. They are also given the opportunity to identify any issues or concerns that they may have with IVRS. Through IVRS, participants will be informed that the IVRS supplements, but do not replace, existing clinical services and that all emergencies should be handled by usual means. If the respondent does not wish to continue in the IVRS arm, they will have the option to move out of this arm and continue in the control arm.

The participants in the control group receive standard care; they will be explained the importance of glucose testing in pregnancy and regular follow-ups as scheduled in the study (Table 1). Baseline sociodemographic information will be collected from participants who discontinue.

**Table 1:** Interventions for the pregnant mothers and its target behavioral correlates

Content of intervention	Operationalized as	Target Behavioral correlate
Mother and Baby Workshop(MBA)	Workshops on healthy diet, GDM, immunization, seminars, token of appreciation and a meal for the parents	Participants turning up for OGTT,follow up at delivery and 3.5 months
Interactive voice response system (IVRS)	IVRS calls to participants for reminding OGTT, follow up visit, child immunization. Regular IVRS call providing health advice to pregnant women and mothers on diet and child care	Participants turning up for OGTT, follow up at delivery and 3.5 months
Control arm	Providing routine care; study information sheet and the research assistant will provide the date of next visit for follow-up	Participants turning up for OGTT, follow up at delivery and 3.5 months

### Outcome measures

#### The primary outcome is the proportion of participants visiting the hospital for OGTT before 36 weeks of gestation

OGTT requires overnight fasting of minimum 8–12 h; the pregnant women will have to come early morning to the research hospital after which the fasting venous sample will be drawn. The research staff will administer 82.5 g of glucose (equivalent to 75 g of anhydrous glucose) mixed with 250 ml of water. Blood will be drawn again after 2 h, and the blood sugar level is measured. Successful follow-up assessment means that the pregnant woman has completed OGTT before the end of 36 gestational weeks. The response rate is the number of women completing the OGTT divided by the total number of women who were due for undergoing OGTT within  36 weeks of gestation. The loss to follow up is defined as the number of women who missed OGTT divided by the total number of women who completed 36 weeks of gestation. The eligibe duration for undergoing OGTT is 14 weeks, starting from 22 weeks till the end of 36 gestational weeks.

#### Secondary outcome measure

1. **The proportion of women who complete the follow-up visits at the time of delivery.** Those who complete the follow-up assessment within 1 week of delivery. Successful follow-up assessment is when the follow-up questionnaire and anthropometry within the designated time of 1 week from the date of delivery is complete. Follow-up rate is defined as the numbers of follow-ups done by research staff divided by the total number of eligible live births in the corresponding period in the study population. The loss to follow up rate will be measured as the number of missed follow-ups divided by the total number of eligible live births due in the corresponding period in the study population. The eligible duration of follow-up for this measure is around 18 weeks, calculated from the time of undergoing OGTT to 40 gestational weeks or at the time of delivery.
2. **The proportion of women who complete the 3.5 months follow up visit:** Successful follow-up assessment is defined as completion of follow-up questionnaire and anthropometry of mother and child within 3.5 months since the date of birth. Follow-up rate will be estimated as the number of follows ups done by research staff divided by the total number of 3.5-month-old infants eligible for follow-up during the corresponding study period in the study population. The loss to follow up rate will be measured as the number of missed follow-ups divided by the total number of 3.5-months-old infants during the corresponding study period in the study population. The time frame for the denominator is 32 weeks, calculated from the 22 weeks of gestation till the infant is 3.5 months old.
3. **Cost-benefit analysis of the IVRS and MBA workshop**

An economic evaluation of the intervention package in improving successive health visits compared to the control group will be done. A cost-effectiveness analysis by assessing the balance between costs of the intervention and effects of improved visits from a healthcare provider perspective. Costs for all separate actions and time used by all individual health care professionals including the costs towards the workshop program, costs for the IVRS and all other materials. The costs involved in the intervention package and the benefits achieved compared with those of control arm will be estimated. With this data, we aim to perform a cost-effective analysis of the interventions after the 3.5-month follow-up.

Data Collection and storage: The research assistants will conduct and collect the interview with the help of an android application; MAASTHI- which was developed specifically for this project. The MAASTHI system aims to collect, validate, verify, and store the data of respondents, their follow-up, and their children. It is an integrated system that consists of a modular design at the core-system which ensures the utmost flexibility without compromising the operational strength. The system comprises a web front end and an Android app. The app is designed to work with the disconnected data model as well as a fully synchronous model. The system is designed with (Secure Sockets Layer) encryption with Secure Hash Algorithm 2 (SHA2). This ensures data privacy and protection. The system employs role-based authentication system. All the interview information will also be printed on paper and stored for documentation.

### Sample size

The current rate of screening is 77% for OGTT, and the expected rate is an increase of 10% through the interventions. In order to achieve 80% power to detect a small improvement of 10% in the screening with a significance level of 5% and 95% confidence interval, the required sample size is 50 in each arm after adding a non-response rate of 30%.

For the secondary objective, we have assumed that 50% will be followed-up at delivery. In order to achieve 80% power to detect a small improvement of 20% in follow up at delivery, with a two-sided significance level 95%, the sample size required in each arm is 104 persons [27, 28]. Assuming refusal and loss to follow up at 50%, the total number of women required per arm is 156 at delivery. Assuming higher loss to follow-up at 70% for following up children at 3 months of age, the required sample size in each arm is 265. Hence, we aim to recruit 265 pregnant women in each arm making a total of 795.

### Statistical analysis

Descriptive statistics of participants’ baseline characteristics will be presented to assess their comparability. These statistics will be reported as a mean (SD) or median (first quartile, third quartile) for continuous variables, and count (per cent) for categorical variables. Baseline characteristics will include age, education, occupation, income, parity, gestational age, and mobile phone access. We calculate the response rate for OGTT in each of the study arms. For the primary analysis, we will compare the proportion of participants retained during the eligible period in the MBA and IVRS group with those in the control group using a Chi-square test.

## Discussion

Earlier evidence suggests that the relationship between the patient and the study personnel is an essential factor in subject accrual and continued participation. E.g., patients who received the nursing interventions based on self-regulation theory experienced less disruption in their usual life activities during and following radiation therapy [29]. Similarly, IVRS too has been gaining acceptance in a variety of studies, ranging from symptom and mood monitoring to adverse event reporting and medication compliance [30]. Additionally, IVRS is used in various populations, including lower socioeconomic profile and difficult-to-reach subjects such as those recovering from drug abuse, economically disadvantaged drinkers with AIDS, and low-income public clinic patients [31, 38, 37]. Study participants using IVRS have reported a high degree of user satisfaction, noting that it is both user-friendly and convenient. However, one real limitation for all IVR systems is that the vast majority of intended users ignore them. Thus, the feasibility of using IVRS on a large scale to address health problems is another critical issue to study [36]. Studies involving counselling and antenatal sessions have been successful in encouraging women to breastfeed [32], increasing energy and protein intake among pregnant women [33] and general antenatal information provision [34, 35].

Studies have to continually innovate and try newer interventions in order to sustain participation. Earlier evidence indicates that use of behavioural intervention was effective in improving health outcomes [11] such as increasing retention in HIV care among pregnant mothers [12], improved contraceptive use [13], to improve medication compliance [14] for the regular follow-up of children and adolescents in psychiatric care [15]. IVRS and telecommunication technology has been effectively used to improve maternal and child health care by providing regular information [16, 17]. Using an intervention to prevent the loss to follow up in a birth cohort is a unique initiative. The validity concerns in cohort studies necessitate this.

## Abbreviations

ANC: Ante-natal checkup
ASHA: Accredited Social Health Activist
AWW: Anganwadi Worker
GDM: Gestational Diabetes Mellitus
HIV: Human Immunodeficiency Virus
IEC: Institutional Ethics Committee
IIPH-H: Indian Institute of Public Health-Hyderabad
IVRS: Interactive voice response system
MAASTHI: Maternal antecedents of adiposity and studying the transgenerational role of hyperglycemia and insulin
MBA: Mother and baby affairs
NFHS: National Family Health Survey
OGTT: Oral Glucose Tolerance Test
RCT: Randomized Controlled Trial
RID: Respondent identification number
SD: Standard deviation
